# Randomized Clinical Trial on the Efficacy of Triple Therapy Versus Sequential Therapy in Helicobacter pylori Eradication

**DOI:** 10.7759/cureus.24897

**Published:** 2022-05-10

**Authors:** Zain Sharif, Muaz Mubashir, Mehdi Naqvi, Hassan Atique, Saira Mahmood, Muneeb Ullah

**Affiliations:** 1 Gastroenterology, Nishtar Hospital Multan, Multan, PAK; 2 Internal Medicine, Federal Government Polyclinic Hospital, Islamabad, PAK; 3 Internal Medicine/Gastroenterology, Federal Government Polyclinic Hospital, Islamabad, PAK; 4 General Surgery, Maroof International Hospital, Islamabad, PAK

**Keywords:** h. pylori, antimicrobial, eradication therapy, stool antigen, randomized trial, sequential therapy, triple therapy, helicobacter pylori

## Abstract

Introduction:* Helicobacter pylori *(*H. pylori*) colonization is prevalent all over the world, and it is associated with low socioeconomic status, poor hygiene, and overcrowding. Its eradication is important since it is an etiologic agent for gastritis, peptic ulcer, gastric carcinoma, and mucosa-associated lymphoid tissue lymphoma. Different regimens are available for the eradication of *H. pylori* and include triple therapy and sequential therapy. Our study aims to compare the efficacy of triple therapy versus sequential therapy in the eradication of *H. pylori*.

Material and methods: This randomized clinical trial was conducted at the Pakistan Institute of Medical Sciences Hospital, Islamabad, from September 2016 to September 2017 after the approval of the institutional review board. A total of 160 patients were enrolled and equally divided into two, group A and group B. A twice-daily dose of amoxicillin 1,000 mg, rabeprazole 20 mg, and clarithromycin 500 mg was given to group A for 10 days, while group B was initially given rabeprazole 20 mg and amoxicillin 1,000 mg two times daily for the first five days (i.e., induction phase), followed by triple therapy that included rabeprazole 20 mg, clarithromycin 500 mg, and metronidazole/tinidazole 500 mg twice daily for the next five days. A negative stool antigen test performed four weeks after the completion of therapy was considered an effective eradication. A proforma was used to collect data that included age, gender, city or province of residence, family income, group (group A or group B), and eradication efficacy. Analysis of the data was performed using the Statistical Package for the Social Sciences version 17 (SPSS Inc., Chicago, USA).

Results: A total of 160 patients were included, with mean age and standard deviation of 40.02±24.4 years. The male/female ratio was 1.8:1. Successful eradication of *H. pylori* achieved in group A was 67.5% (N=54) in comparison to group B, which was 95% (N=76) (p=0.001).

Conclusion: Sequential therapy was superior to triple therapy in *H. pylori* eradication.

## Introduction

*Helicobacter pylori* (*H. pylori*) colonizes over 50% of the world population and about 70%-90% of the population in developing countries [1,2]. Moreover, early acquisition of *H. pylori*, which often persists lifelong, is attributed to low socioeconomic status, poor hygienic practices, and overcrowding [2]. It often remains asymptomatic without any complications in up to 85% of individuals [3,4]. Furthermore, it is an etiologic agent for a variety of upper gastrointestinal diseases with significant morbidity [5]. Its association is well established with chronic active gastritis, atrophic gastritis, duodenal ulcer, gastric ulcer, gastric adenocarcinoma, and mucosa-associated lymphoid tissue lymphoma [3,6]. *Helicobacter pylori* can be diagnosed by endoscopic histology, biopsy urease test, culture test, and polymerase chain reaction test of the gastric biopsy specimen. Noninvasive tests include detection by fecal antigen assay, serology, and urea breath test [4,7]. The stool antigen test has a sensitivity and specificity of 85%-95% [4]. The increasing prevalence of antimicrobial resistance in *H. pylori* from person to person has led to the failure of eradication therapy [8,9]. To avoid drug resistance, a combination of antibiotics is used instead of a single antibiotic [4]. Many international guidelines recommend triple therapy (clarithromycin, amoxicillin or metronidazole and proton pump inhibitor (PPI), or ranitidine bismuth citrate) for 7-14 days as the first line of treatment for its eradication [8,10]. A second-line therapy and antimicrobial treatment based on culture from a gastric biopsy are reserved for failure of first-line therapy [8]. Despite a large number of studies, the single best therapeutic regimen for the treatment of *H. pylori* could not be established. In trials, sequential therapy has shown higher eradication rates than standard triple therapy [11,12]. However, management guidelines differ among countries and depend on local susceptibility patterns [13]. A local study in Pakistan reported a high clarithromycin resistance responsible for decreased *H. pylori* eradication with triple therapy [14]. Our study aims to compare the efficacy of sequential therapy versus triple therapy in the eradication of *H. pylori*.

## Materials and methods

This randomized clinical trial was conducted in the Department of Gastroenterology of Pakistan Institute of Medical Sciences Hospital, Islamabad, from September 2016 to September 2017. The ethical approval letter numbered F. 1-1/2015/ERB/SZABMU was taken before the commencement of the trial from Shaheed Zulfiqar Ali Bhutto Medical University. A simple random sampling technique was used. A total of 160 patients presenting in the gastroenterology department were enrolled for this randomized clinical trial after obtaining informed consent. Patients above the age of 18 years who tested positive for *H. pylori* by stool antigen test were included in the study. Patients with a history of proton pump inhibitors or antibiotics (within four weeks before stool antigen testing), gastric carcinoma, gastric surgery, chronic liver or renal disease, chronic diarrhea, pregnancy, lactation, drug abuse, and poor compliance to medications were excluded from the study. The patients were divided into two groups using the lottery method, group A and group B, each containing 80 patients. A twice-daily dose of amoxicillin 1,000 mg, rabeprazole 20 mg, and clarithromycin 500 mg was given to group A for 10 days, while group B was initially given rabeprazole 20 mg and amoxicillin 1,000 mg two times daily for the first five days (i.e., induction phase), followed by triple therapy that included rabeprazole 20 mg, clarithromycin 500 mg, and metronidazole/tinidazole 500 mg twice daily for the next five days. The eradication course was followed four weeks later by a repeat stool antigen test. A negative result was considered an effective eradication of *H. pylori*. Those who tested positive were given an alternative treatment. A proforma was used to collect data that included age, gender, city or province of residence, family income, group (group A or group B), and eradication efficacy. Patient confidentiality was maintained. Analysis of the data was performed using the Statistical Package for the Social Sciences version 17 (SPSS Inc., Chicago, USA). The frequency and percentage for qualitative variables included efficacy and gender. Standard deviation and mean were calculated for the age of the patients. Stratification was used to affect modifiers such as age and gender. Post-stratification chi-square test was used. Its value of less than 0.05 with a 95% confidence interval was considered statistically significant.

## Results

A total of 160 patients detected with *H. pylori* were included, with a mean age and standard deviation of 40.02±24.4 years. The minimum age was 18 years, while the maximum was 65 years. Male predominance was seen with 64.6% compared to females who were 35.4%. The male/female ratio was 1.8:1. Age distribution in terms of frequency and percentage is shown in Table 1.

**Table 1:** Age distribution of patients

Age category	Number	Percentage
18-20	3	1.8
21-30	25	15.6
31-40	32	20
41-50	47	29.3
51-60	41	25.6
61 and above	12	7.5

Most of the patients were residents of the federal capital of Islamabad, followed by Punjab and Khyber Pakhtunkhwa. The geographical and socioeconomic distribution is shown in Table 2.

**Table 2:** Geographical and socioeconomic parameters of patients PKR: Pakistani Rupee

Parameter		Number	Percentage
Place of residence	Islamabad (federal capital)	82	51.2
	Punjab	29	18.1
	Khyber Pakhtunkhwa	49	30.6
Family income per month	Less than 10,000 PKR	58	36.25
	More than 10,000 PKR	102	63.5

Successful eradication of *H. pylori* achieved in group A was 67.5% (N=54) in comparison to group B, which was 95% (N=76) (p=0.001). Stratification according to gender and age is shown in Table 3.

**Table 3:** Stratification according to gender and age

Parameter		Triple therapy		Sequential therapy		P-value
		Eradication	Number (%)	Eradication	Number (%)	
Gender	Male (n=103)	Yes	40 (78.43)	Yes	48 (94.1)	0.00031
		No	22 (22.52)	No	3 (5.8)	
	Female (n=57)	Yes	14 (49.1)	Yes	28 (98.2)	0.00051
		No	13 (45.6)	No	2 (7)	
Age	18-20 (n=3)	Yes	2 (60)	Yes	3 (100)	0.000190
		No	0 (0)	No	0 (0)	
	21-30 (n=25)	Yes	12 (48)	Yes	9 (36)	0.000231
		No	6 (24)	No	0 (0)	
	31-40 (n=32)	Yes	12 (37.5)	Yes	16 (50)	0.00043
		No	4 (12.5)	No	0 (0)	
	41-50 (n=47)	Yes	19 (40.42)	Yes	12 (25.5)	0.000623
		No	13 (27.6)	No	1 (2.1)	
	51-60 (n=41)	Yes	6 (14.6)	Yes	28 (68.2)	0.000511
		No	3 (7.3)	No	2 (4.8)	
	61 and above (n=12)	Yes	3 (25)	Yes	8 (66.6)	0.000111
		No	0 (0)	No	1 (8.33)	

## Discussion

The average presenting age in our study was 40.0±24.4 years. This was similar to a local study in Lahore with 40.51±13.04 years and an international study in Qatar with 38.85±11.78 years [11,15]. Male predominance was seen in our study, which is consistent with a local study [16]. A significant proportion (36.25%) of the population belonged to very low socioeconomic status with a monthly income of roughly 54 US dollars. This is very low considering that the majority of patients are residents of the federal capital. Additionally, many of them live in single room houses with average of five members in a family. Overcrowding along with a poor hygienic environment and lack of basic facilities is directly related to the increased prevalence of *H. pylori* [2]. The etiologic association of *H. pylori* with variety of gastrointestinal diseases mandates eradication therapy. This not only helps in the prevention of disease but also lessens the cost burden and morbidity and increases the quality of life. In our study, successful eradication of *H. pylori* with triple therapy was seen in 67.5% of the patients, while successful eradication with sequential therapy was seen in 95% of the patients. This was in accordance with other studies that showed sequential therapy to be more effective than triple therapy [17-19]. The efficacy of triple therapy is decreasing, and the culprit held responsible is clarithromycin resistance [20]. Still, sequential therapy is not the first-line therapy for eradication as previous studies were unable to reach optimal results [21]. Additionally, the choice of second-line therapy after failure of sequential therapy is yet to be standardized. On the contrary, there are other studies in which sequential therapy was less effective [11,22,23]. The decreased efficacy of sequential therapy is secondary to dual resistance associated with metronidazole and clarithromycin [24]. Modifications in management guidelines are thus based on the locoregional efficacy of regimens and antimicrobial resistance. The main factors affecting the regimen used and its efficacy include compliance of the patient, antimicrobial resistance, drug brands, side effects of drugs, complex drug regime, especially in the case of sequential therapy, and geographical area. Hence, while deciding the therapy regimen, one must take these parameters into account. There were no significant side effects reported in our study. The stool antigen test for detecting *H. pylori* is noninvasive and has good specificity and sensitivity [25]. Additionally, it was performed in nearly all laboratories in regional area; therefore, it was used for the detection of *H. pylori *in our study. Its cheap cost makes it affordable for private patients and lessens the burden on the government for those who had their tests performed at our institute.

Our study lacked data on antimicrobial sensitivity and resistance, which is an important factor before starting the therapy and determining its efficacy. However, it adds to the cost burden. Our study was limited to a small proportion of patients. We did not standardize the brand names of medications that were used in the study. Different brands have variable quality, cost, and efficacy. We also did not standardize the laboratories for stool antigen testing. Both of these can affect the results.

## Conclusions

Sequential therapy is superior to triple therapy in our community across all ages and gender for the eradication of *H. pylori*. The stool antigen test is a noninvasive and effective test in determining the eradication of *H. pylori*.
